# ERp29 controls invasion and metastasis of gastric carcinoma by inhibition of epithelial-mesenchymal transition via PI3K/Aktsignaling pathway

**DOI:** 10.1186/s12885-017-3613-x

**Published:** 2017-09-06

**Authors:** Jianxin Ye, Jinsheng Huang, Jie Xu, Qiang Huang, Jinzhou Wang, Wenjing Zhong, Xinjian Lin, Yun Li, Xu Lin

**Affiliations:** 1 Key Laboratory of Ministry of Education for Gastrointestinal Cancer, School of Basic Medical Sciences, Fujian Medical University, 1 Xueyuan Road, Minhou, Fuzhou, Fujian 350108 China; 2 Department of Gastrointestinal Surgery, The First Affiliated Hospital of Fujian Medical University, Fuzhou, China; 3 Fujian Key Laboratory of Tumor Microbiology, Fujian Medical University, Fuzhou, China

**Keywords:** ERp29, Gastric cancer, Epithelial-mesenchymal transition, PI3K, AKT

## Abstract

**Background:**

Gastric cancer (GC) accounts for the fourth most occurring malignancy and the third major cause of cancer death. Identifying novel molecular signaling pathways participating in gastric tumorigenesis and progression is pivotal for rational design of targeted therapies to improve advanced GC outcome. Recently, the endoplasmic reticulum (ER) protein 29 (ERp29) has been shown to inversely associate with primary tumor development and function as a tumor suppressor in breast cancer. However, the role of ERp29 in GC patients’ prognosis and its function in GC progression is unknown.

**Methods:**

Clinical importance of ERp29 in the prognosis of GC patients was assessed by examining its expression in 148 GC tumor samples and correlation with clinicopathological characteristics and survival of the patients. The function and underlying mechanisms of ERp29 in GC growth, invasion and metastasis were explored both in vitro and in vivo.

**Results:**

Downregulation of ERp29 was commonly found in GC tissues and highly correlated with more aggressive phenotypes and poorer prognosis. Functional assays demonstrated that knockdown of ERp29 increased GC cell migration and invasion and promoted metastasis. Conversely, ectopic overexpression of ERp29 produced opposite effects. Mechanistic studies revealed that loss of ERp29 induced an epithelial-to-mesenchymal transition (EMT) in the GC cells through activation of PI3K/Akt pathway signaling.

**Conclusion:**

These findings suggest that downregulation of ERp29 is probably one of the key molecular mechanisms responsible for the development and progression of GC.

**Electronic supplementary material:**

The online version of this article (10.1186/s12885-017-3613-x) contains supplementary material, which is available to authorized users.

## Background

While recent decades have witnessed therapeutic advances, the clinical outcome of gastric cancer (GC) is still disappointing in view of the facts that a majority of GC patients has advanced to late stage at diagnosis and that current chemotherapy only offers limited survival advantage.

GC is a very aggressive malignancy representing the third leading cancer mortality worldwide [1]. Advanced stage at initial diagnosis of GC is commonly seen in a large percentage of GC patients presenting unresectable disease or distant metastases. Moreover, it is one of the most challenging clinical tasks to effectively manage and treat advanced GC patients. Conventional systemic chemotherapy has limited efficacy for advanced GC cancer with only a minority of the patients achieving a satisfactory response [2]. Thus, there is certainly a need to identify novel biomolecules for possible GC early diagnosis, prognosis prediction and potential targets for development of novel therapeutic agents that target such pivotal molecular signaling pathways participating in gastric carcinogenesis and progression.

The endoplasmic reticulum (ER) protein 29 (ERp29) is expressed ubiquitously and abundantly in eukaryotic cells and normally serves as a molecular chaperone in protein secretion from the ER [3–5]. Protein structural analysis demonstrates that N-terminal domain of ERp29 involves dimerization essential to its function in unfolding and escort of secretory proteins [6] while the C-terminal domain is necessary for substrate binding and secretion [7, 8].

ERp29 biological and pathological functions in carcinogenesis of epithelial cancers were in controversy. Several studies reported that ERp29 functioned as a tumor suppressor since its expression suppressed tumor formation in mice [9, 10] and was inversely correlated with tumor development in breast, lung and gallbladder cancer [11, 12]. In contrast, ERp29 expression could also sustain tumor cell survival against genotoxic insults by chemotherapy and radiation therapy [13–15]. Intriguingly, ERp29 is found to be involved in inducing mesenchymal–epithelial transition (MET) of cancer cells and epithelial morphogenesis implicating its another important role in predisposing cancer cells to survival and metastasis as well [9, 11]. Regardless, a functional link between ERp29 expression and GC progression has yet to be described. In this study, we evaluated the expression of ERp29 in primary GC tumors and analyzed its prognostic significance in the GC patients. In addition, we explored the function of ERp29 in GC growth and invasion in vitro and metastasis in vivo. We report here that loss of ERp29 expression was commonly observed in GC and strongly correlated with poor clinical outcome. Knocking down ERp29 promoted GC cell invasion and metastasis. Mechanistically, ERp29 suppression promoted EMT in GC cells as evidenced by a profound reduction of E-cadherin and ZO-1 expression, an increase of Snail and Twist expression and an activation of the PI3K/AKT pathway.

## Methods

### Clinical samples and immunohistochemical analysis

Human GC samples and their adjacent nontumorous gastric tissues were obtained from surgical resection performed at the First Affiliated Hospital of Fujian Medical University (Fuzhou, China) during the period of 2008 to 2010. The resected specimens were either frozen in liquid nitrogen and stored at −80 °C freezer immediately or fixed in 10% formalin for paraffin embedding. Written informed consent was obtained from all patients according to the Declaration of Helsinki and this study had been approved by our institutional review board and regulatory authorities. Clinicopathological classification and staging were determined by the standards of American Joint Committee on Cancer (AJCC) Seventh Edition of GC TNM Staging [16]. Tissue cores were extracted from 148 gastric tumors for construction of a tissue microarray (TMA) with at least two tissue cores per sample of 1 mm diameter. A rabbit anti-ERp29 monoclonal antibody (1:100, Abcam, ab42002) was used for immunohistochemical staining of formalin-fixed, paraffin-embedded tissue sections cut from TMAs. To assess the degree of nuclear or cytoplasmic staining, a 5-tiered scale was employed according to the average percentage of positively stained cells. Value 0, 1, 2, 3, 4 represented ≤5%, 5% -25%, 26%–50%, 51%–75% and ≥75% positive cells, respectively. Assigned values were then multiplied with the staining intensity of 0 (no staining), 1 (weak staining, light yellow), 2 (moderate staining, yellow brown), or 3 (strong staining, brown) to obtain a score ranging from 0 to 12. A score equal to or less than 3 was considered low expression of ERp29, and a score greater than 3 was considered high ERp29 expression.

### Western blot analysis

Western & IP cell lysis buffer (Beyotime, Shanghai, China) with PMSF (Amresco, Solon, Ohio, USA) was used to lyse tissues or cells for 30 min on ice following centrifugation at 12000 g for 10 min at 4 °C. BCA Protein Assay Kit (Thermo Scientific, Waltham, MA, USA) was employed to measure total proteins in the supernatants collected from the centrifugation. The equal amount of proteins were separated on 10% SDS-PAGE and transferred to a 0.45 μm PVDF membrane (Amersham Hybond, GE Healthcare, München, Germany) followed by blocking in 0.5% albumin from bovine serum (Amresco, Solon, Ohio, USA) overnight at 4 °C with specific antibodies. The primary antibodies used in the study were as follows: rabbit anti-ERp29, rabbit anti-pan-AKT and mouse anti-β-actin diluted at 1:2000 (Cell Signaling Technology, Danvers, MA, USA); rabbit anti-E-cadherin, rabbit anti-ZO-1, rabbit anti-snail, rabbit anti-Ser473-AKT, rabbit anti-Thr308-AKT, rabbit anti-GSK-3β, rabbit anti-phospho-GSK-3β(Ser9), rabbit anti-mTOR and rabbit anti-phospho-mTOR all diluted at 1:1000 (Cell Signaling Technology); rabbit anti-Vimentin diluted at 1:1500 (Abcam, ab92547). After 3 times washing in TBST for 10 min each time, the membrane was then incubated with the respective secondary antibodies at room temperature for 1 h and the immunoblot was developed through enhanced chemiluminescence (Lulong biotech, Xiamen China).

### RNA extraction and quantitative real-time PCR

RNA in cultured cells or frozen tissues was extracted using Trizol reagent (Ambion, Carisbad CA,USA) and reverse transcribed to cDNA by RT Reagent Kit (TaKaRa, Dalian, China). Quantitative real-time PCR was carried out in Mx3000P QPCR system (Agilent Technologies, Santa Clara, CA, USA) by using SYBR Premix EX Taq kit (Takara, Shiga, Japan). Primers for human Slug, Snail, E-cadherin and Vimentin were designed (Additional file 1: Table S1) for measuring the relative mRNA expression of the respective genes by the 2^-△△ct^ method after normalization to endogenous β-actin.

### Cell lines

Human gastric cancer cell lines SGC7901 (NTCC411001) and MGC803 (NTCC411124) were obtained from the Type Culture Collection of the Chinese Academy of Sciences (Shanghai, China) and cultured in RPMI-1640 (Gibco) medium supplemented with 10% FBS (Gibco) at a humidified atmosphere of 5% CO_2_ at 37 °C.

### Plasmids and generation of stable GC cell lines

Opening reading frame of human ERp29 gene was PCR-amplified and inserted into lentiviral expression vector pCDH-CMV-MCS-EF1-RFP-Puro (System Biosciences, Mountain View, CA, USA). The resulting plasmid or empty vector without insert was co-transfected with lentiviral packaging plasmids pMDL, pVSVG and pRev into 293 T cells. 48 h post co-transfection the supernatants were collected for infecting MGC803 or SGC7901 cells cultured in 6-cm dishes. The clones surviving from puromycin selection were expanded into cell clones as being ERp29 over-expressing cells (MGC803-pERp29 or SGC7901-pERp29), or empty control cells (MGC803-pCDH or SGC7901-pCDH). For generation of ERp29 knocking down clones, shERp29 fragment was cloned into pSuper-retro-puro plasmid (Oligoengine, Seattle, Washington, USA) and the resulting recombinant plasmid or empty vector with no inserts was co-transfected into 293 T cells with lentiviral packaging plasmids pIK (Invitrogen Carlsbad, CA). The supernatants collected from the co-transfection culture were used to infect MGC803 or SGC7901 cells. The clones resistant to puromycin were expanded into the cell clones as being ERp29 knockdown cells (MGC803-pshERp29 or SGC7901-pshERp29), or empty control cells (MGC803-pSuper or SGC7901-pSuper). ERp29 expression levels in these cell lines were evaluated by western blot analysis. The sequences of the primers and oligonucleotides used are listed in Additional file 1: Table S1.

### Cell proliferation assay

Cells were seeded into 96-well plate at a density of 2 × 10^3^ cells per well and incubated for 24, 48, 72, 96 or 120 h. The proliferation of cells was evaluated by the Cell Counting Kit-8 (CCK-8, Dojindo, Kuma-moto, Japan). 10 μl CCK-8 reagent was added into each well and incubated for 4 h. The absorbance from each well was determined using a microplate reader at the wavelength of 450 nm (Bio-Tek, Winooski, VT, USA).

### Colony formation assay

5 × 10^2^ cells were grown in 60-mm plate containing complete growth medium for 14 days and the colonies formed that contained 50 or more cells were counted after staining with crystal violet for 5 min. For the soft agar colony formation assay, the cell suspension containing 5 × 10^3^ in 2 × DMEM with 20% FBS were mixed with equal volume of 0.7% agarose and immediately laid on top of an underlayer of 0.5% agarose made in 1× DMEM supplemented with 10% FBS. The plates were cultured for 5 to 21 days when the surviving colonies (>50 cells per colony) were counted and photographed with a Qimaging micropublisher 5.0 RTV microscope camera (Olympus, Tokyo, Japan).

### Cell migration and invasion assay

For the migration assay, 4 × 10^4^ cells suspended in serum-free media were plated onto the upper chamber of Transwell insert (8-mm pore size; BD Bioscience). As for the invasion assay, equal cells were plated onto the Transwell insert coated with Matrigel (BD Bioscience). The medium supplemented with 20% FBS in the lower chamber functioned as chemoattractant. 24 h after incubation at 37 °C the cells in the upper surface of chambers were removed with cotton swab and then the cells that successfully migrated or invaded through the pores and located on the lower surface of filter were stained with 0.1% crystal violet in 20% methanol, photographed, and counted using a Qimaging Micropublisher 5.0 RTV microscope camera (Olympus).

### Wound-healing assay

Cells were grown to nearly 100% confluence in 6-well plate and scratch was made through the cell monolayer by a 200 μl disposable pipette tip. After washing three times with HBSS, the cells were cultured in fresh growth medium and incubated for 0, 24 or 48 h at which point wound closure was photographed, respectively.

### In vivo metastasis study

Cell suspension containing 2 × 10^6^ MGC-ERp29 or MGC-vector cells in 0.2 ml serum-free RPMI-1640 was prepared and injected intravenously via the lateral tail vein in female BALB/c nude mice. 12 weeks after injection, all mice were euthanized and the lungs and liver were resected. Metastasis on the lungs and liver was thoroughly examined under dissecting microscope and using histopathologic analysis. The in vivo studies were approved by the Fujian Medical University Institutional Animal Care and Use Committee.

### Immunofluorescent staining

For immunofluorescent staining, cells were seeded onto 8-μm-thick section slides and fixed in 4% ice-cold paraformaldehyde for 10 min after overnight culturing. Afterwards, the cells were blocked with 10% normal goat serum (ZSGB Biotech, China) for 10 min and incubated with antibodies against Vimentin and E-cadherin overnight at 4 °C. On the next day, cells were washed three times and incubated with Alexa Fluor 488 conjugated goat anti-rabbit secondary antibody (1:200, 2 mg/ml, Invitrogen). DAPI (2 mg/ml, Invitrogen) was used to counterstain the nuclei and cells were visualized with a laser scanning confocal microscope (Leica, Germany).

### Statistical analysis

SPSS 17.0 for Windows was used to perform statistical analysis and all data were expressed as mean ± SD from 3 separate assays. Pearson’s chi-square test and Spearman’s rank-order correlation were employed to analyze an association between ERp29 expression and the clinicopathological parameters. Kaplan-Meier analysis was performed to plot the survival curves. Differences were considered significant when *p* values were smaller than 0.05.

## Results

### ERp29 downregulation in GC is correlated with poor prognosis

**Fig. 1:**
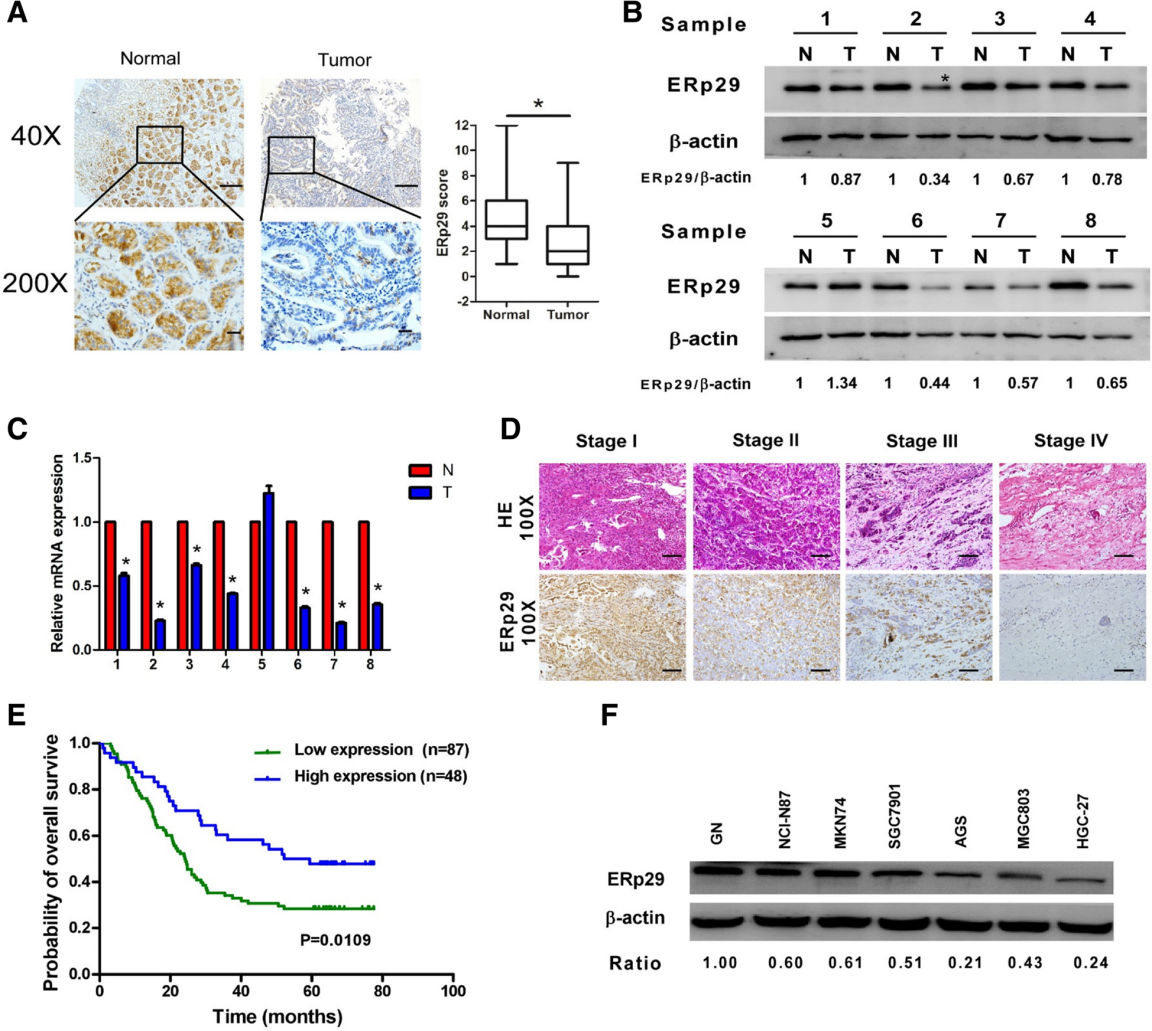
ERp29 was downregulated in gastric carcinoma and inversely correlated with prognosis. (**a**) Representative images of IHC staining of GC tissues and adjacent normal tissues (40 × magnification; scale bar: 50 μm; 200 × magnification; scale bar: 20 μm). (**b**) Western blot analysis of ERp29 expression in 8 pairs of gastric tumor (T) and adjacent non-tumorous mucosa (N). (**c**) ERp29 mRNA expression level in the eight pairs of gastric tumor (T) and adjacent normal mucosa (N). *P < 0.05. (**d**) Representative HE and ERp29 immunohistochemical staining of different clinical stages of gastric cancers (100 × magnification; scale bar: 50 μm). (E) Kaplan-Meier survival analysis showing that downregulation of ERp29 in GC was associated with the patients’ poorer overall survival

To discern the prognostic relevance of ERp29 expression, IHC was performed in a cohort of archived tumor samples from 148 gastric cancer patients. As shown in the representative Fig. 1a, significantly lower ERp29 expression was seen in the primary GC tumors than in the adjacent normal tissues. The lower expression of both ERp29 protein (Fig. 1b) and mRNA level (Fig. 1c) was also confirmed in the gastric tumor tissues as compared with the adjacent normal tissues by western blot analysis and qRT-PCR. The Pearson *χ2* test and Spearman’s rank-order correlation analysis of ERp29 expression with clinicopathologic features demonstrated that low-level expression of ERp29 in GC tissues was correlated with advanced clinical stage (Fig. 1d and Table 1). Caution might be excised that only small patient cohort was included in the correlation study, therefore, larger numbers of patients would be required to draw more clinically relevant conclusions. Nevertheless, given the observation that ERp29 expression was downregulated in GC, Kaplan–Meier analysis was employed to evaluate the relationship of ERp29 protein expression as assessed by IHC with patient outcome. As shown in Fig. 1e and f, patients with tumors expressing low ERp29 had significantly shorter survival than patients with tumors that expressed high levels of ERp29. Collectively, these data suggest that ERp29 may serve as a tumor suppressor and its downregulation may promote GC development and progression.

**Table 1:** Clinicopathological characteristics of 148 GC patients according to ERp29 expression

Characteristic	ERp29		*P* value*	*r*_s_ value
	Low expression (*n* = 96)	High expression (*n* = 52)		
Normal vs cancer				
Normal	47	101	<0.001	−0.331^#^
Cancer	96	52		
Age(years)				
<60	36	16	0.413	0.059
≥ 60	60	36		
Gender				
Females	31	8	0.026	-
Males	65	44		
TNM stage				
II	21	27	0.001	−0.286^#^
III	66	22		
IV	9	3		
TNM stage				
I, II	21	27	<0.001	−0.306^#^
III, IV	75	25		
T classification				
T2	6	5	0.011	−0.237^#^
T3	32	29		
T4	58	18		
N classification				
N0	13	12	0.007	−0.257^#^
N1	15	18		
N2	20	8		
N3	48	14		
Lymphatic metastasis				
Yes	83	40	0.139	−0.121
No	13	12		
Distant metastases				
Yes	9	3	0.541	−0.063
No	87	49		
Venous invasion				
Yes	48	20	0.179	−0.066
No	87	49		
Locattion				
Up	24	13	0.179	0.134
Middle	34	8		
Lower	30	26		
Pathological differentiation				
Un−/Poorly	70	33	0.233	−0.111
Moderrately/Well	26	19		
Perineural invasion				
Yes	26	19	0.233	0.111
No	70	33		

*P value was determined using Pearson’s chi-square test; *r*_s_ value was determined by Spearman’s rank-order correlation, ^#^: *P* < 0.05

### Effect of ERp29 on GC cell proliferation, migration, invasion and metastatic potential

**Fig. 2:**
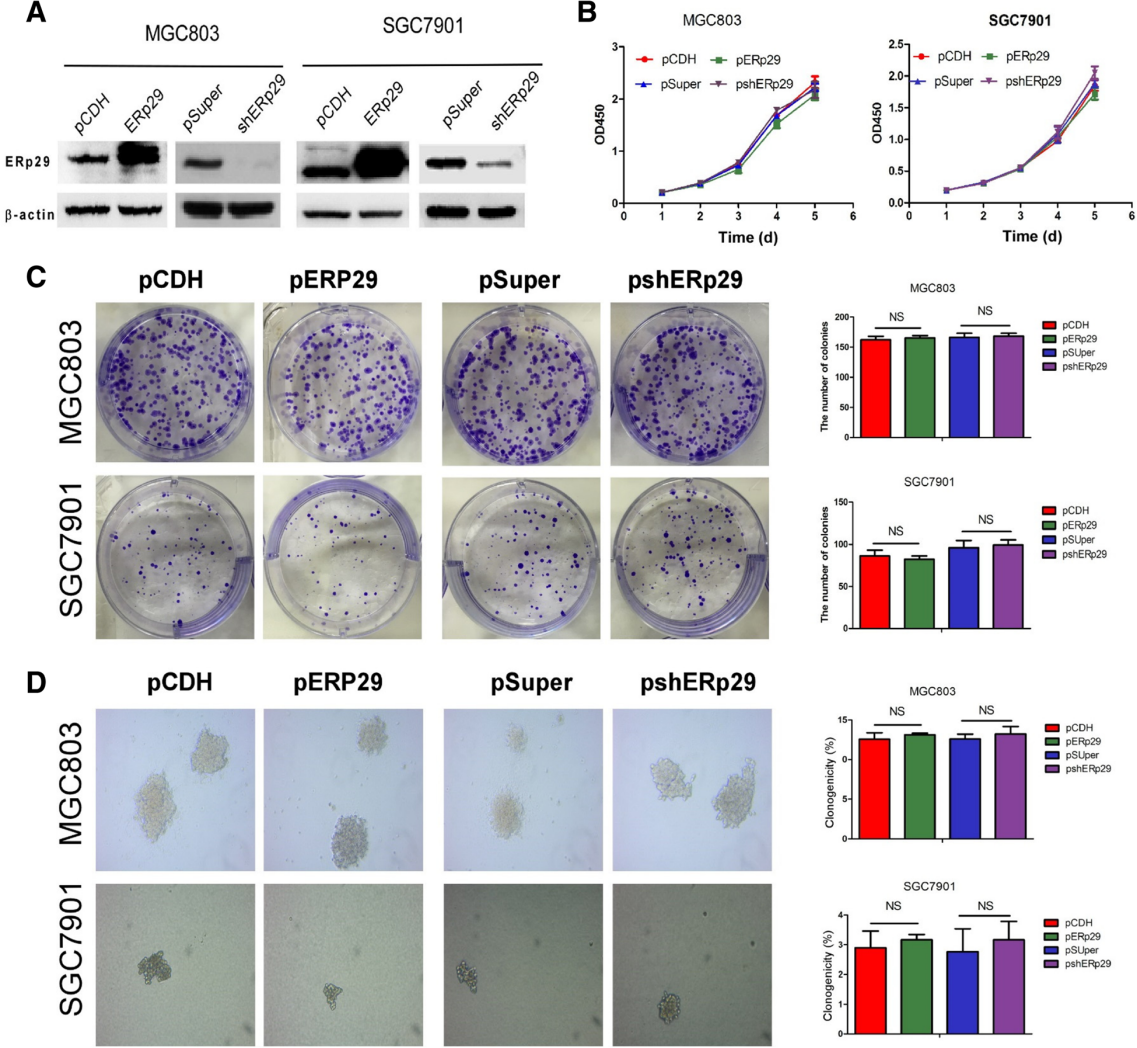
ERp29 had no effect on GC cell proliferation. (**a**) Western blot analysis confirming expression of ERp29 in ERp29 overexpressed or knocked down MGC803 and SGC7901 stable cell lines. (**b**) CCK-8 assay. (**c**) Colony formation assay. (**d**) Soft agar colony formation assay

Given that ERp29 expression is of prognostic significance in GC, we examined how ERp29 functionally regulates GC malignant behaviors both in vitro and in vivo. Both genetic silencing and overexpression approaches were taken to specifically knock down or overexpress ERp29 in the GC cell lines MGC803 and SGC7901. Western blot analysis confirmed stable overexpression or knockdown of ERp29 in these cells (Fig. 2a). Overexpression or knockdown of ERp29 did not produce any change in the rate of proliferation of MGC803 or SGC7901 cells in vitro as assessed by CCK-8 assay (Fig. 2b), colony formation assay (Fig. 2c), and soft agar colony formation assay (Fig. 2d).

**Fig. 3:**
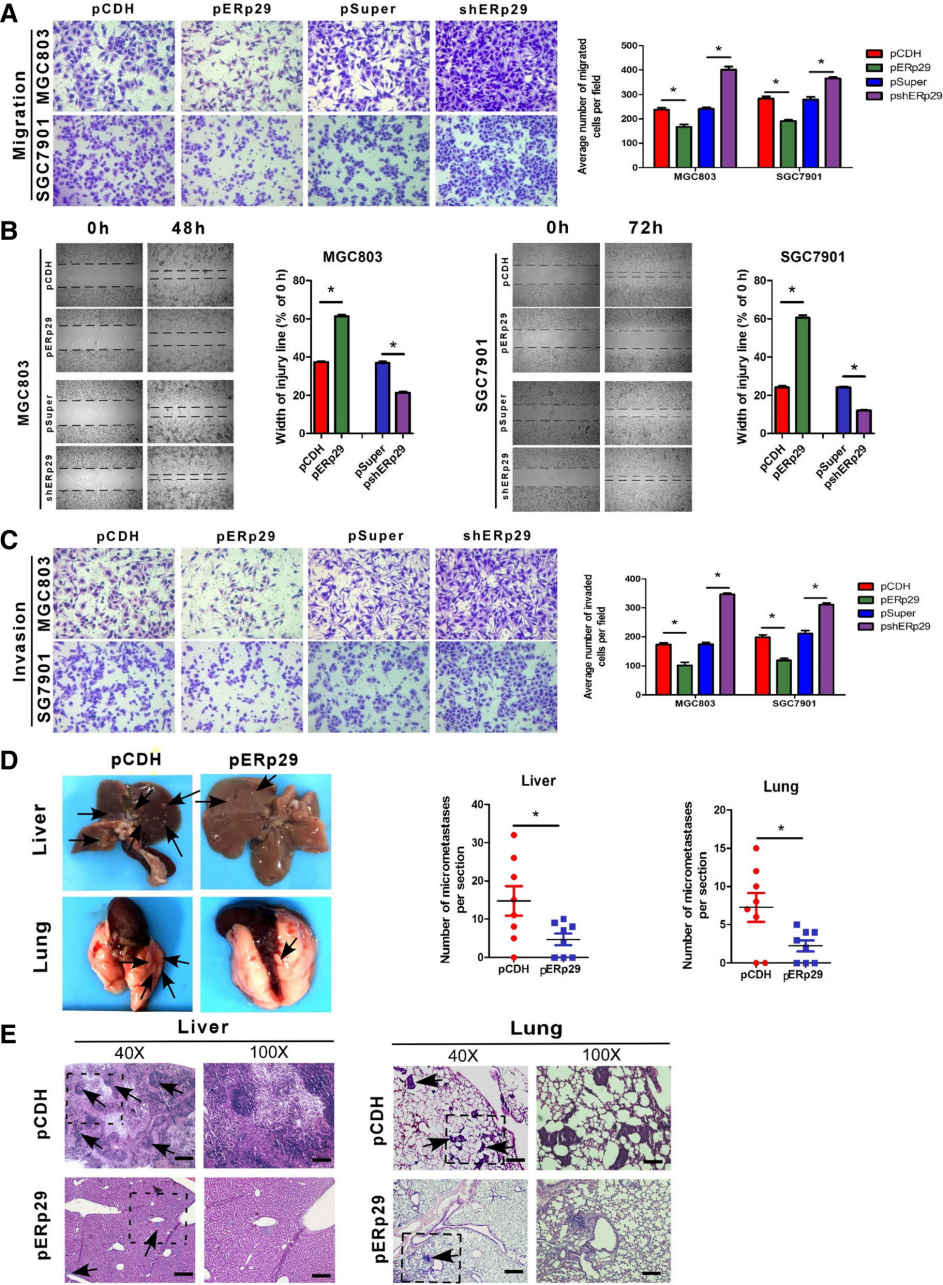
ERp29 regulated GC cell migration, invasion and metastatic potential. (**a**) Relative migration of the GC cells through an uncoated filter toward serum-containing medium in a Boyden chamber assay. (**b**) Relative motility as determined by the ability of the GC cells to close a wound made by creating a scratch through a lawn of confluent cells. (**c**) Relative invasion of cells through a layer of Matrigel coated on the filter of a Boyden chamber. (**d**) Quantification of liver and lung metastatic burden in mice 10 weeks after tail vein injection of the GC cells by counting the number of micrometastases per section. (**e**) Hematoxylin and eosin staining of fixed and paraffin-embedded tissues confirmed the presence of micrometastases in the liver (40 × magnification; scale bar: 50 μm) and lungs (100 × magnification; scale bar: 20 μm) of mice injected with the GC cells. *P < 0.05

We then compared the influence of ERp29 on cell migration by a Boyden two chamber assay where the cells were attracted by FBS on the other side of chamber to migrate. As shown in Fig. 3a, migration was suppressed by overexpression of ERp29 but enhanced when ERp29 was knocked down. Then we performed a wound-healing/scratch assay in order to confirm the cell migratory ability as wound closure is a generally accepted measure of cell motility. Fig. 3b showed that the ERp29-overexpressing cells slowed down the cell migration as compared to the empty-vector transfected control cells whereas the wound closure was faster in ERp29-knockdown cells than in the scrambled shRNA control cells. A modified Boyden chamber invasion assay was used to determine cell invasive capacity in the context of ERp29 expression by quantifying the number of cells invading through Matrigel layer at 48 h or 72 h after the cells were plated on Matrigel-coated transwell inserts. As expected, overexpression of ERp29 in these GC cells significantly reduced their invasive potential (Fig. 3c). In contrast, knockdown of ERp29 in MGC803 and SGC7901 caused a significant increase of their invasive ability. The influence of ERp29 on in vivo metastatic potential was evaluated by injecting 2 × 10^6^ MGC803-ERp29 or control MGC803-pCDH cells into the tail vein of BALB/c nude mice. 10 weeks after injection, mice were sacrificed and metastatic nodules were counted on the sections of the liver and lungs. As shown in Fig. 3d and e, the livers and lungs of the mice injected with ERp29-overexpressing MGC803-ERp29 cells had much fewer nodules formed as compared to the mice injected with the control MGC803-pCDH cells. Taken together, these results clearly suggest that ERp29 functions to restrain migration, invasion and metastasis as well.

### ERp29 regulates EMT process in GC cells

**Fig. 4:**
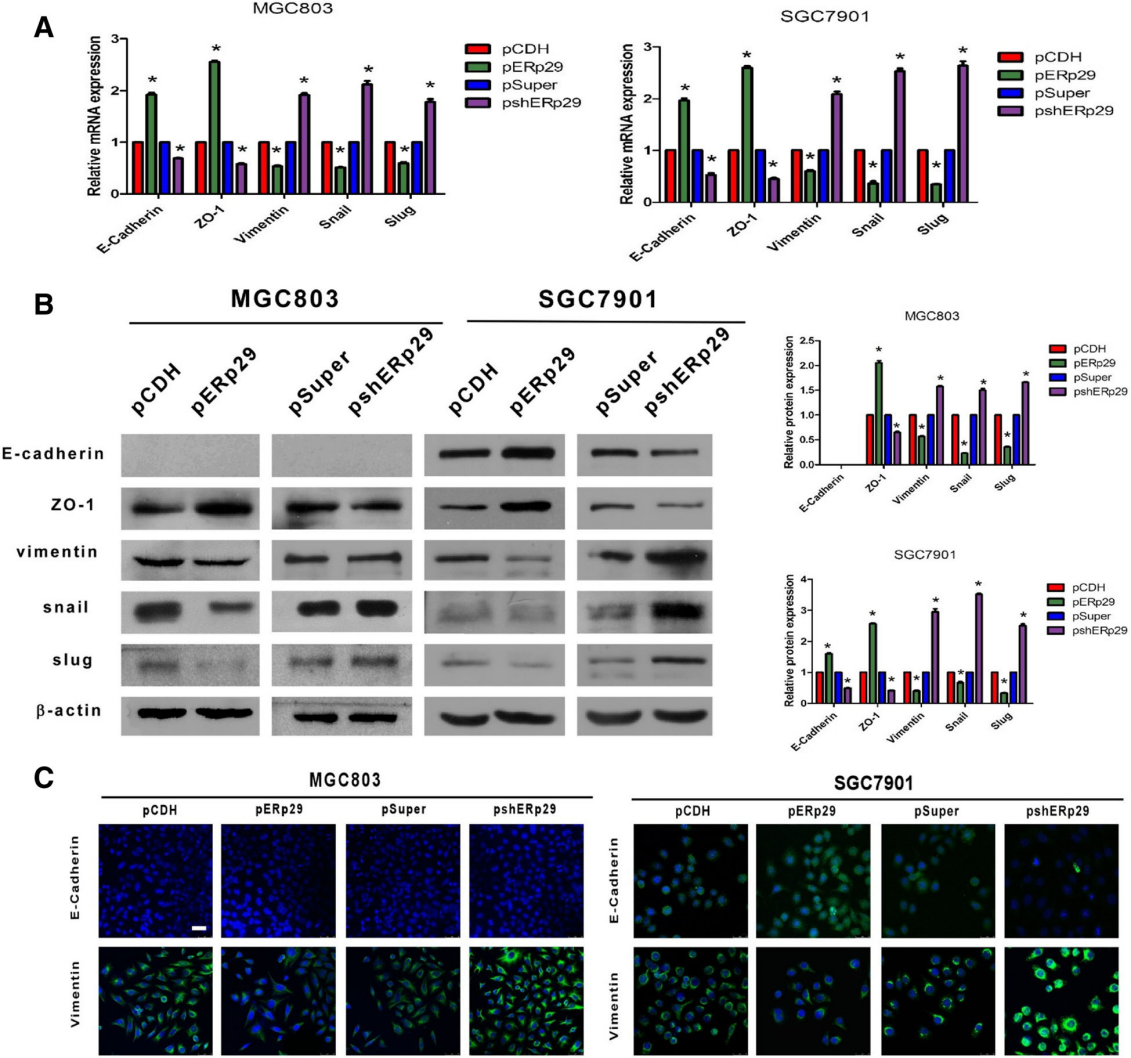
ERp29 regulated the expression of EMT markers in the GC cells. (**a**) qRT-PCR analysis of the expression of EMT markers in the ERp29 over-expressed or knockdown MGC803 and SGC7901 cells. (**b**) Western blot analysis of the expression of EMT markers in the ERp29 over-expressed or knockdown MGC803 and SGC7901 cells. (**c**) Immunofluorescent staining of E-cadherin and Vimentin expression in the ERp29 over-expressed or knockdown MGC803 and SGC7901 cells (400 × magnification; scale bar: 25 μm). *P < 0.05

Initiation of EMT in cancer cells is a key step in the metastatic process, endowing them with motile and invasive properties. ERp29 modulating EMT has been observed in breast cancer cells [9, 11]. Thus, we sought to determine whether ERp29 also participates in regulation of EMT process in the gastric cancer cells. To this end, the expression of EMT-associated markers in the ERp29 overexpressing or knockdown MGC803 and SGC7901 cells was quantified. As shown in Fig. 4a, ERp29 overexpression resulted in an increase of mRNA expression of epithelial biomarkers (E-cadherin and ZO-1) but a decrease of the expression of both mesenchymal marker (Vimentin) and EMT transcription factors (Snail and Twist). In sharp contrast, knockdown of ERp29 led to the opposite effect as reflected by a significant reduction in E-cadherin and ZO-1, and a marked increase in Vimentin and Snail and Twist. Western blot analysis also disclosed a similar pattern of expression for those EMT markers although E-cadherin was undetectable in the MGC803 cells (Fig. 4b). Furthermore, confocal microscopy study confirmed the enhanced expression of E-cadherin but the decreased expression of Vimentin in the ERp29 overexpressed cells whereas ERp29 knockdown in the GC cells caused a reduction in E-cadherin expression but an increase in Vimentin expression (Fig. 4c). These findings suggest that knockdown of ERp29 promotes GC cell migration and invasion through activation of an EMT process.

### ERp29 suppresses PI3K/Akt signaling

**Fig. 5:**
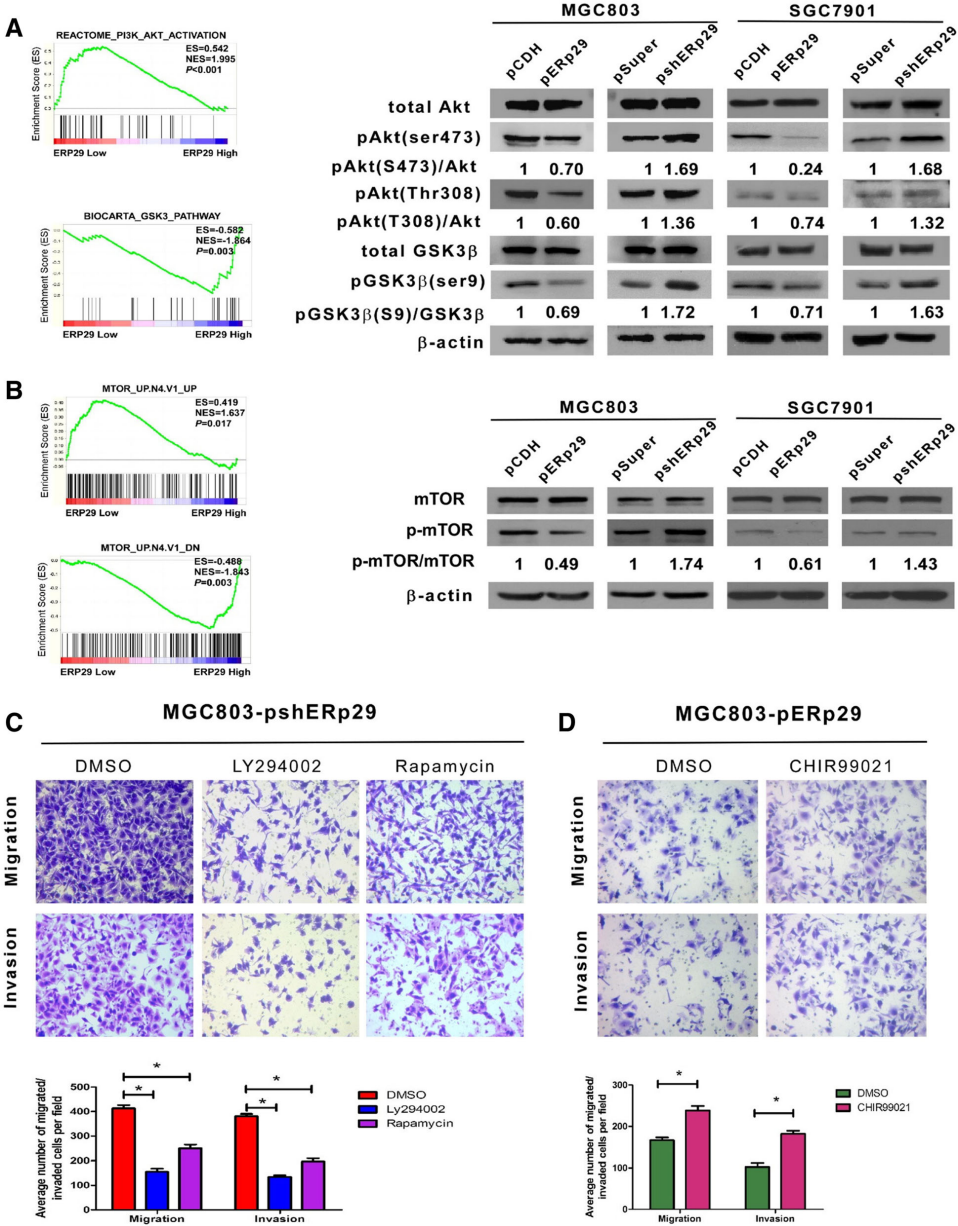
Regulation of EMT by ERp29 was dependent on PI3K/Akt pathway. (**a**) GSEA plot showing that ERp29 expression negatively correlated with Akt-activated gene signatures (REACTOME_PI3K_AKT_ACTIVATION) and positively correlated with GSK3β-pathway (BIOCARTA_GSK3_PATHWAY) (left panel). Western blot analysis of total Akt, pAKT(Ser473), pAKT(Thr308), GSK-3β and pGSK3β(Ser9) expression in the indicated gastric cancer cell lines. (**b**) GSEA plot showing that ERp29 expression negatively correlated with Akt/mTOR pathway (MTOR_UP.N4.V1_UP, MTOR_UP.N4.V1_DN). Western blot analysis of mTOR, p-mTOR expression in indicated gastric cancer cell lines. (**c**) LY294002 and rapamycin reversed the effect of ERp29 overexpression on the cell migratory and invasive abilities. (**d**) CHIR99021 reversed the effect of ERp29 knockdown on the cell migratory and invasive abilities. *P < 0.05

To understand the molecular mechanisms by which ERp29 induced the inhibition of GC malignant progression, the bioinformatic algorithms of gene set enrichment analysis (GSEA) through The Cancer Genome Atlas (TCGA) database were employed to predict ERp29-related signaling pathway-regulated gene signatures. It was found that expression levels of ERp29 in tumor specimens were negatively correlated with the PI3K/Akt/GSK3β and Akt-mTOR signaling pathway-activated gene signatures and positively correlated with the PI3K/Akt/GSK3β and Akt-mTOR signaling pathway suppressed gene signatures (Fig. 5a and b, left panels). To verify these predicted results, we dissected the activity of this pathway in the context of ERp29 by examining the phosphorylation status of Akt, GSK3β and mTOR, the downstream effectors of PI3K. As shown in Fig. 5a and b (right panels), upregulation of ERp29 in MGC803 and SGC7901 cells decreased p-AKT(Ser473), p-AKT(Thr308) and p-GSK3β(Ser9) levels while ERp29 knockdown significantly increased phosphorylation of Akt, GSK3β and mTOR. To confirm and extend the results of the western blot analyses, we treated the ERp29 overexpressed or knockdown GC cells with PI3K/Akt inhibitor LY294002, GSK inhibitor CHIR99021 and allosteric mTOR inhibitor rapamycin respectively, then measured the effect of such pharmacological inhibition on the GC cell migration and invasion. As shown in Fig. 5c, Akt or mTOR inhibition by LY294002 or rapamycin partially abrogated the effect of ERp29 knockdown enhanced cell migration and invasion. Treatment of ERp29 overexpressed MGC803-pERp29 cells with GSK inhibitor CHIR99021 increased their migratory and invasive capabilities (Fig. 5d). These data suggest that ERp29 serves to restrain motility and invasiveness of the GC cells via a pathway involving PI3K/Akt/GSK3β or Akt-mTOR signaling.

## Discussion

The results obtained from this study provide several lines of evidence supporting that the expression of ERp29 influences the behavior of GC. We showed that ERp29 was downregulated in primary GC tumors and the downregulation of ERp29 was associated with tumor stage and grade as well as poor survival of the patients. In the experimental models, specific knockdown of ERp29 in the GC cells enhanced migration, invasion and metastatic colonization of the lungs and liver. Knockdown of ERp29 in the epithelial GC cells promotes acquisition of EMT traits via activation of PI3K/Akt signaling pathway. These findings imply that ERp29 is likely to functionally serve as a tumor suppressor and that its loss promotes EMT and tumor progression.

EMT is one of the most significant biologic processes in the initial invasion step during cancer metastasis, which allows polarized epithelial cells to become irregular mesenchymal cells. After a series of biochemical changes that induce a morphological transformation, epithelial cells reduced intercellular adhesion and enhanced migratory and invasive capabilities [17–20]. A hallmark of EMT is characteristic of reduced expression of the epithelial markers E-cadherin and ZO-1 but elevated expression of the mesenchymal marker Vimentin. Meanwhile, transcriptional modulators such as Snail, Slug, Twist and β-catenin were up-regulated in EMT [21–23]. ERp29 induced EMT has been found in basal-like MDA-MB-231 breast cancer cells [11]. Consistently, we found the upregulation of E-cadherin but downregulation of Vimentin when ERp29 was stably overexpressed in the GC cell lines. In contrast, knockdown of ERp29 endowed the GC cells with EMT characteristics exemplified by loss of E-cadherin, upregulation of mesenchymal marker Vimentin, and enhanced expression of E-cadherin transcription repressors Snail and Twist.

A central feature of EMT is manifested by activation of the PI3K/Akt pathway in tumor cell lines and clinical samples [24]. AKT participates in a wide array of oncogenic processes such as cell growth and survival, cell cycle progression, metabolism and EMT [11]. In the case of the Akt-induced EMT, the cell is characterized by loss of apico-basolateral cell polarization and cell–cell adhesion, increased cell motility but decreased cell–matrix adhesion, and alterations in the distribution or production of specific markers [25]. Akt usually becomes highly activated by its phosphorylation at both the Thy308 and Ser473 sites, which would decrease the expression of E-cadherin and the tight junction protein ZO-1 in cancer cells, increase metastasis in vivo, induce EMT [25, 26]. An important contribution of PI3K/Akt activation to the aggressive phenotypes of tumor cells is further supported by the observation that the PI3K inhibitor LY294002 can reverse these effects [27]. In line with this, we found that PI3K/Akt activation is a characteristic feature of the GC cells that have undergone an EMT. Although it is not clear whether a defective ERp29 is the sole determinant of the invasive phenotype, the capability of forced expression of ERp29 in reversal of the invasive phenotype and the enhancement of Akt and mTOR phosphorylation triggered by loss of ERp29 both indicate that ERp29 may play a critical role in this process. GSK3β is the kinase primarily responsible for phosphorylating β-catenin for degradation. GSK-3β phosphorylation by Akt on Ser9 inhibits its activity and prevents β-catenin from degradation [28, 29]. A significant increase in the level of Ser9-phosphorylated and therefore inactive GSK-3β was found in the ERp29 knockdown cells implicating coordinate activation of Wnt/β-catenin signaling pathway while the exact mechanism of how ERp29 suppresses β-catenin activity remains to be elucidated. It may be noteworthy that GSK3β could regulates Snail and Slug phosphorylation for degradation respectively through β-Trcp-mediated ubiquitination and the CHIP pathway [30, 31].

Intriguingly, while we have demonstrated that ERp29 depletion induces a significant enhancement of the GC cell migration and invasion by acquisition of EMT traits through activation of PI3K/Akt signaling pathway, it does not affect the proliferation rate of the cells. PI3K/Akt signaling is well known for its capacity to promote tumor progression by activating cell proliferation and growth as well as metastasis. However, it has also been reported that inhibition of PI3K/Akt in breast, non-small cell lung and gastric cancer cells restrains tumor invasion and metastasis independent of its growth inhibitory effects [32–34]. Although the detailed mechanism is yet to be expounded, our observation that knockdown of ERp29 has a strong influence on GC cell migration and invasion rather than direct effects on proliferation further supports the notion that processes governing tumor metastasis downstream of PI3K/Akt may be separable from those driving proliferation.

## Conclusions

In summary, this is the first study to date demonstrating that ERp29 may functionally serve as a tumor suppressor in GC. Downregulation of ERp29 in GC tumors and its strong correlation with tumor progression and patients’ prognosis merit further efforts to develop it as a diagnostic and prognostic biomarker. Furthermore, ERp29 may be exploited as a potential target in cancer therapy since it regulates PI3K/Akt and β-catenin pathways, which are well-established and recognized signaling events associated with malignant transformation.

## Additional file

Additional file 1: Table S1. Oligonucleotides used for cloning and qRT-PCR. (DOCX 28 kb)

## Abbreviations

EMT: Epithelial-to-mesenchymal transition
ERp29: Endoplasmic reticulum protein 29
GC: Gastric cancer
GSEA: Gene set enrichment analysis
MET: Mesenchymal–epithelial transition
qRT-PCR: Quantitative real-time PCR
TCGA: The Cancer Genome Atlas
TMA: Tissue microarray
