# Supplementary material for: ERp29 controls invasion and metastasis of gastric carcinoma by inhibition of epithelial-mesenchymal transition via PI3K/Aktsignaling pathway
Source: BMC Cancer. 2017 Sep 6;17:626. doi: 10.1186/s12885-017-3613-x (PMC5585903; doi:10.1186/s12885-017-3613-x)
Supplement: Additional file 1: Table S1. — Oligonucleotides used for cloning and qRT-PCR. (DOCX 28 kb) [file 12885_2017_3613_MOESM1_ESM.docx]

Additional 1: Table S1. Oligonucleotides used for cloning and qRT-PCR

| **Oligonucleotides** | **Sequences (5′-3′)** |
| --- | --- |
| **shERp29 cloning** | |
| shERp29 sense strand | GATCCCAACTCGGCTTCCAGCGATGATTCAAGAGATCATCGCTGGAAGCCGAGTTTTTTTA |
| shERp29R antisense strand | AGCTTAAAAAAACTCGGCTTCCAGCGATGATCTCTTGAATCATCGCTGGAAGCCGAGTTGGG |
| **ERp29 cloning** |  |
| ERp29 F | CGGAATTCGCCACCATGGCTGCCGCTGTGCCCCG |
| ERp29 R | CGGGATCCTTACTTGTCGTCATCGTCTTTGTAGTCCAGCTCCTCTTTCTCGG |
| **Real-time RT-PCR** | |
| β-actin F | CCTGGCACCCAGCACAAT |
| β-actin R | GGGCCGGACTCGTCATACT |
| Snail F | CCTTCTCTAGGCCCTGGCT |
| Snail R | AGGTTGGAGCGGGTCAGC |
| Slug F | GACACATTAGAACTCACACGG |
| Slug R | TACACAGCAGCCAGATTCCT |
| E-cadherin F | GTCTGTCATGGAAGGTGCT |
| E-cadherin R | TACGACGTTAGCCTCGTTC |
| Vimentin F | CCACGAAGAGGAAATCCAGG |
| Vimentin R | CAGAGAGGTCAGCAAACTTGG |
